# Associations between single and multiple dietary vitamins and the risk of periodontitis: results from NHANES 2009–2014

**DOI:** 10.3389/fnut.2024.1347712

**Published:** 2024-04-08

**Authors:** Feizhao Liang, Mei Lu, Yanping Zhou

**Affiliations:** ^1^Department of Stomatology, Liuzhou People’s Hospital, Liuzhou, Guangxi, China; ^2^Department of Prosthodontics, Affiliated Stomatology Hospital of Guilin Medical University, Guilin, Guangxi, China

**Keywords:** multiple vitamins co-exposure, periodontitis, Bayesian kernel machine regression, weighted quantile sum, quantile g-computation model

## Abstract

**Background:**

Periodontitis is a prevalent inflammatory periodontal disease that has an impact on the overall quality of life. Although several studies have indicated an association between individual vitamin intake and periodontitis risk, the associations of the multivitamins with periodontitis risk remain unclear.

**Aim:**

This study aimed to explore the joint effect of multivitamins (including vitamin A, vitamin B_1_, vitamin B_2_, vitamin B_6_, vitamin B_12_, vitamin C, vitamin D, vitamin E, and vitamin K) on periodontitis.

**Methods:**

For this cross-sectional study, data were collected from participants aged ≥ 30 years in the National Health and Nutrition Examination Surveys 2009–2014 (*n* = 9,820). We employed weighted multivariate logistic regression models to evaluate the single association between individual vitamin intakes and periodontitis, and Bayesian kernel machine regression (BKMR), weighted quantile sum (WQS) regression, and quantile g-computation (qgcomp) models to assess the joint effect of nine vitamins on periodontitis.

**Results:**

The overall prevalence of periodontitis was approximately 35.97%. After adjustment of covariates, vitamin B_6_ [odds ratio (OR) = 0.82, 95% confidence interval (CI): 0.72–0.94] and vitamin E (OR = 0.79, 95%CI: 0.69–0.92) were negatively related to the likelihood of developing periodontitis, respectively. The result of three models indicated that, mixture of vitamin A, vitamin B_1_, vitamin B_2_, vitamin B_6_, vitamin B_12_, vitamin C, vitamin D, vitamin E, and vitamin K had a significant negative combined effect on the risk of periodontitis. In the BKMR model, when all remaining vitamins were at their median levels, the periodontitis risk decreased with increased concentration levels of vitamin E and vitamin B_2_. WQS analysis indicated the highest weighted chemical was vitamin E, followed by vitamin B_12_ and vitamin D. In the qgcomp model, vitamin E received the highest negative weights for the periodontitis risk, followed by vitamin B_2_ and vitamin D, respectively.

**Conclusion:**

Both dietary vitamin B_6_ and vitamin E were associated with decreased odds of periodontitis. Additionally, the mixture-exposed analyses consistently showed the negative correlations between nine dietary vitamins mixtures and periodontitis.

## Introduction

Periodontitis is a prevalent inflammatory periodontal disease that constitutes the primary etiology of tooth loss in adults (1). In addition to its impact on oral health, periodontitis has been linked to an increased susceptibility to chronic conditions such as cardiovascular disease (CVD) (2), prediabetes (3), Alzheimer’s disease (4), and pre-eclampsia (5). Therefore, it holds immense significance to identify modifiable influencing factors for the prevention and management of periodontitis.

Nutrition is recognized as a key modifiable factor for periodontitis, and maintaining a well-balanced nutrition is crucial for promoting periodontal health (6). Vitamin, which is crucial element for maintaining normal physiological functions in the human body, can be obtained from the diet and nutritional supplements (7). A number of epidemiological studies have found the association between vitamin and periodontitis risk. For example, vitamin C and vitamin E, can actively participate in the oxidative stress process within the body and exert an anti-inflammatory effect by eliminating reactive oxygen species. As a result, they effectively prevent the occurrence and progression of periodontitis (8, 9). A survey based on National Health and Nutrition Examination Survey (NHANES) data revealed a non-linear relationship between dietary vitamin C intake and periodontitis risk, and both too low and high vitamin C intake were associated with an elevated periodontitis (10). A recent study investigated the association between different vitamin intake and periodontitis risk, revealing a negative correlation between levels of vitamin A and vitamin B_2_ intake and periodontitis risk. Conversely, excessive consumption of vitamin B_1_ may elevate the risk of developing periodontitis (11). However, to the best of our knowledge, most current nutritional epidemiological studies solely evaluate the impact of individual vitamin intake on periodontitis (9–11), rather than considering co-intakes of all vitamins. In general, populations are typically exposed to multiple dietary vitamins in combination, which may result in synergistic, or antagonistic effects (12).

Therefore, this study aims to explore the joint effect of multivitamins on periodontitis based on NHANES database, including vitamin A, vitamin B_1_, vitamin B_2_, vitamin B_6_, vitamin B_12_, vitamin C, vitamin D, vitamin E, vitamin K, which provided certain references for the diet management of periodontal health.

## Methods

### Study participants

This cross-sectional study used data from NHANES database 2009–2014. NHANES is a nationally representative survey of the non-institutionalized U.S. population, gathering data through self-reported questionnaires, laboratory assessments and clinical examinations (13). The NHANES only collects periodontal examination data for individuals aged over 30 years between 2009 and 2014. The NHANES database was reviewed and approved by the Ethics Review committees of both the Centers for Disease Control (CDC) and the National Center for Health Statistics (NCHS) (Protocol Number: NHANES 2009–2010: Protocol #2005–06; NHANES 2011–2014: Protocol #2011–17.). The requirement of ethical approval for this was waived by the Institutional Review Board of Liuzhou People’s Hospital, because the data was accessed from NHANES (a publicly available database). The need for written informed consent was waived by the Institutional Review Board of Liuzhou People’s Hospital due to retrospective nature of the study.

**Figure 1:**
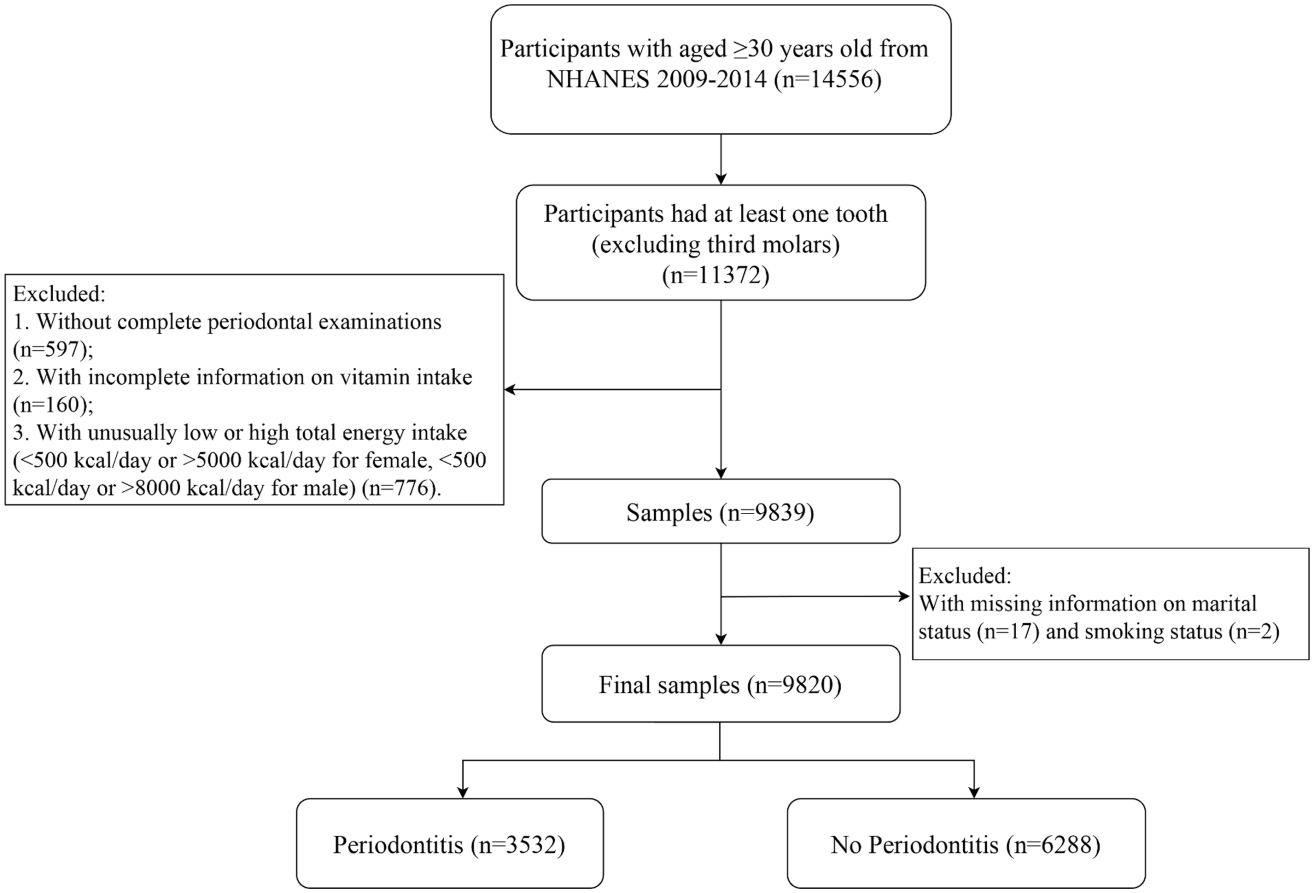
Flow chart for the selection of participants in the study.

For the purpose of this study, we included participants aged≥30 years old who had at least one tooth (excluding third molars) (*n* = 11372). Notably, the exclusion criteria were listed as follows: (1) Participants who did not undergo complete periodontal examinations. (2) Participants with incomplete information on vitamin intake. (3) Participants with unusually low or high total energy intake (<500 kcal/day or > 5000 kcal/day for female, <500 kcal/day or > 8000 kcal/day for male). (4) Participants with missing information on key co-variables. The analysis ultimately encompassed a total of 9820 subjects. The detailed flow chart of this study is depicted in Figure 1.

### Exposure and outcome variables

For each NHANES participant, the NHANES database employs a 24 h dietary recall method to gather information on participants’ diets (12). In this study, vitamins included vitamin A, vitamin B_1_, vitamin B_2_, vitamin B_6_, vitamin B_12_, vitamin C, vitamin D, vitamin E, and vitamin K. Vitamins intake levels were assessed by both dietary intake and dietary supplement intake. The total daily intake of vitamins was obtained by summing the first 24 h dietary recall interviews and the daily intake of dietary supplements. Each dietary vitamin in this study is divided into two categories according to the weighted median.

Periodontitis was considered as the primary outcome in this study. Clinical examinations of periodontal and dental status were conducted by dental examiners for NHANES participants aged ≥30 years between 2009 and 2014. Used the criteria proposed by Eke et al. (14), periodontitis was categorized into three groups: mild, moderate, and severe. Periodontitis was regarded as mild if there were ≥ 2 interproximal sites with attachment loss (AL) ≥3 mm, and ≥ 2 interproximal sites with pocket depth (PD) ≥4 mm (not on the same tooth) or one site with PD ≥5 mm. The definition of moderate periodontitis was established as the presence of 2 interproximal sites with AL ≥ 4 mm, not on the same tooth, or the presence of at least 2 interproximal sites with PD ≥5 mm, not on the same tooth. Severe periodontitis was defined as 2 interproximal sites with AL ≥ 6 mm (not on the same tooth), or ≥ 1 interproximal sites with PD ≥ 5 mm (not on the same tooth). In this study, mild, moderate, and severe periodontitis were classified as having periodontitis.

### Covariates

Possible covariates in this study were as follows: age (years), gender (male/female), race/ethnicity (white, black, or other race), education level (<high school, or ≥ high school), marital status (married & living with a partner, or never married& divorced& separated& widowed), poverty-income ratio (PIR, <1, ≥1, or unknown), smoking status (never smoked, former smoker, or current smoker), alcohol consumption (never drinker, moderate drinker, heavy drinker, or unknown), body mass index (BMI, kg/m^2^, <25, 25–30, or ≥ 30), physical activity [metabolic equivalent (MET)·min/week, <450, or ≥ 450], hypertension (No/Yes), diabetes (No/Yes), dyslipidemia (No/Yes), CVD (No/Yes), nonsteroidal anti-inflammatory agents (No/Yes), anti-infectives (No/Yes), serum vitamin D (<50 nmol/L, or ≥ 50 nmol/L), white blood cell (WBC, 1000 cells/uL), decayed teeth (No/Yes), dental floss (No/Yes), total energy (kcal/day), total sugars (g/day), and forms of vitamin A [including Alpha-carotene (μg), Beta-carotene (μg), Beta-cryptoxanthin (μg), Lycopene (μg), Lutein + zeaxanthin (μg)]. Alcohol consumption was determined based on drinking frequency: never drinkers (0 drinks per day), moderate drinkers (<2 drinks per day for males and < 1 drink per day for females), and heavy drinkers (≥2 drinks per day for males and ≥ 1 drink per day for females). BMI category was defined as three levels according to World Health Organization (WHO) guidelines: BMI < 25 kg/m^2^ (underweight & normal), 25 ≤ BMI < 30 kg/m^2^ (overweight), and BMI ≥ 30 kg/m^2^ (obesity).

### Statistical analysis

Three weighted variables were adopted: WTDRD1, SDMVPSU, and SDMVSTRA. The measurement data were reported as Mean ± standard error (Mean ± S.E), and group comparisons were conducted *T*-test or *F*-test. Counting data were presented as the number of cases and proportion of components, with differences between groups assessed Chi-square test. The missing values in this study were imputed using multiple interpolation methods, and a sensitivity analysis was performed on the data before and after interpolation (Supplementary Table S1). First, taking periodontitis as the outcome, we developed weighted univariate logistic regression to identify significant variables (*p* < 0.05), which was used to perform the multivariate logistic regression analysis, and the backward stepwise regression method was employed to further screen the covariates (Supplementary Table S2). Second, we used weighted univariate and multivariate logistic regression models to evaluate the association between individual vitamin intakes and periodontitis. Odds ratio (OR) with 95% confidence interval (CI) were calculated. Lastly, we employed three mixture analysis methods: Bayesian kernel machine regression (BKMR), weighted quantile sum (WQS) regression, and quantile g-computation (qgcomp) models, to assess the joint effect of nine vitamins on periodontitis.

BKMR model-integrating Bayesian and statistical learning approaches to estimate the nonlinear and/or interactive effect in the association between exposure and outcome. Pearson correlation analysis was used to calculate the correlation coefficient between the nine vitamins. Nine vitamins were grouped according to the correlation coefficient diagram. The Group Posterior Inclusion Probability (GroupPIP) and Conditional Posterior Inclusion Probability (CondPIP) quantify the probability of each group, and vitamin of each group being included in the model, indicating their respective contributions to the overall effect. We used R packages “bkmr” to assess the joint effects of nine vitamins on periodontitis risk. Meanwhile, the exposure of single vitamin and periodontitis risk were also obtained.

WQS regression model-assessing the combined effects of nine vitamins co-exposure, and the contributing effects of individual vitamin (15). We used the R package “WQS” to calculate the WQS index comprised of weighted sums of individual vitamin concentrations. The weight assigned to each vitamin in the WQS index reflects its individual contribution to the overall impact.

Quantile g-computation (qgcomp) model-assessing the change in periodontitis risk for a simultaneous one quantile increase in the nine vitamins, irrespective of the direction of correlation between exposures and results (16). The sum of the positive and negative weights is 2. R package “qgcomp” was used to perform the analysis.

In addition, SAS 9.4 and R 4.2.3 software were used for statistical analyses. *p* < 0.05 was considered as statistically significant difference.

## Results

### Basic characteristics of the study subjects

A total of 9,820 subjects were included in this study, with a mean age of 50.95 ± 0.26 years and a male population accounting for 48.91%. Table 1 presents the basic characteristics of the included subjects. 37.52% subjects were obesity. The average total energy for all population was 2158.68 ± 14.42 kcal/day. The overall prevalence of periodontitis is approximately 35.97% (*n* = 3,532) in this study. Compared with the non- periodontitis group, periodontitis group was characterized by a higher age, lower education level, higher rates of hypertension and diabetes history, higher WBC level, total energy, and total sugars (*p* < 0.05).

**Table 1:** Basic characteristics of the study subjects.

Variables	Total (*n* = 9,820)	Non-periodontitis (*N* = 6,288)	Periodontitis (*N* = 3,532)	*p*
Age, years, Mean ± S. E	50.95 ± 0.26	50.61 ± 0.29	51.80 ± 0.41	0.009
Gender, *n* (%)				<0.001
Female	4,967 (51.09)	3,591 (56.01)	1,376 (39.03)	
Male	4,853 (48.91)	2,697 (43.99)	2,156 (60.97)	
Race/ethnicity, *n* (%)				<0.001
White	4,348 (69.14)	3,161 (74.64)	1,187 (55.67)	
Black	2004 (10.46)	1,071 (7.94)	933 (16.64)	
Others	3,468 (20.40)	2056 (17.42)	1,412 (27.69)	
Education level, *n* (%)				<0.001
Less than high school	2,212 (14.73)	1,081 (10.77)	1,131 (24.44)	
High school or above	7,608 (85.27)	5,207 (89.23)	2,401 (75.56)	
Marital status, *n* (%)				<0.001
Married & Living with partner	6,400 (68.79)	4,201 (71.09)	2,199 (63.15)	
Never married &Divorced &Separated &Widowed	3,420 (31.21)	2087 (28.91)	1,333 (36.85)	
PIR, *n* (%)				<0.001
<1	1,688 (11.12)	875 (8.47)	813 (17.60)	
≥1	7,360 (82.70)	4,975 (86.02)	2,385 (74.57)	
Unknown	772 (6.18)	438 (5.51)	334 (7.83)	
Smoking status, *n* (%)				<0.001
Never smoked	5,519 (55.98)	3,828 (60.42)	1,691 (45.10)	
Former smoker	2,481 (26.67)	1,600 (26.90)	881 (26.09)	
Current smoker	1820 (17.36)	860 (12.68)	960 (28.80)	
Drinking status, *n* (%)				0.004
Never drinker	2,418 (19.35)	1,608 (19.74)	810 (18.39)	
Moderate drinker	879 (9.50)	558 (9.88)	321 (8.58)	
Heavy drinker	4,893 (57.12)	3,164 (57.59)	1729 (55.95)	
Unknown	1,630 (14.03)	958 (12.79)	672 (17.08)	
Physical activity, MET· min/week, *n* (%)				0.003
<450	3,510 (32.26)	2,221 (31.20)	1,289 (34.87)	
≥450	6,310 (67.74)	4,067 (68.80)	2,243 (65.13)	
Hypertension, *n* (%)				<0.001
No	4,106 (44.71)	2,782 (46.86)	1,324 (39.43)	
Yes	5,714 (55.29)	3,506 (53.14)	2,208 (60.57)	
Diabetes, *n* (%)				<0.001
No	7,973 (85.84)	5,239 (87.67)	2,734 (81.37)	
Yes	1847 (14.16)	1,049 (12.33)	798 (18.63)	
Dyslipidemia, *n* (%)				0.710
No	2,530 (25.54)	1,644 (25.69)	886 (25.18)	
Yes	7,290 (74.46)	4,644 (74.31)	2,646 (74.82)	
CVD, *n* (%)				0.156
No	7,943 (83.45)	5,096 (83.89)	2,847 (82.39)	
Yes	1877 (16.55)	1,192 (16.11)	685 (17.61)	
Nonsteroidal anti-inflammatory agents, *n* (%)				0.295
No	8,569 (87.71)	5,465 (87.99)	3,104 (87.03)	
Yes	1,251 (12.29)	823 (12.01)	428 (12.97)	
Anti-infectives, *n* (%)				0.032
No	9,368 (94.87)	5,965 (94.47)	3,403 (95.85)	
Yes	452 (5.13)	323 (5.53)	129 (4.15)	
BMI, kg/m^2^, *n* (%)				<0.001
<25	2,593 (27.20)	1757 (28.55)	836 (23.89)	
25–30	3,393 (35.28)	2,181 (35.61)	1,212 (34.46)	
≥30	3,834 (37.52)	2,350 (35.84)	1,484 (41.66)	
Serum vitamin D, nmol/L, *n* (%)				<0.001
<50	2,908 (22.39)	1,608 (18.63)	1,300 (31.60)	
≥50	6,912 (77.61)	4,680 (81.37)	2,232 (68.40)	
WBC, 1000 cells/uL, Mean ± S. E	7.13 ± 0.04	6.97 ± 0.04	7.52 ± 0.07	<0.001
Decayed teeth, *n* (%)				<0.001
No	6,822 (75.47)	4,860 (81.97)	1962 (59.56)	
Yes	2,998 (24.53)	1,428 (18.03)	1,570 (40.44)	
Dental floss, *n* (%)				<0.001
No	3,130 (27.34)	1,665 (23.19)	1,465 (37.51)	
Yes	6,690 (72.66)	4,623 (76.81)	2067 (62.49)	
Total energy, kcal/day, Mean ± S. E	2158.68 ± 14.42	2127.75 ± 15.90	2234.46 ± 24.92	<0.001
Total sugars, g/day, Mean ± S. E	112.34 ± 1.18	110.53 ± 1.51	116.77 ± 1.49	0.003
Vitamin A, μg, *n* (%)				<0.001
<528.00	5,298 (49.96)	3,240 (47.74)	2058 (55.38)	
≥528.00	4,522 (50.04)	3,048 (52.26)	1,474 (44.62)	
Vitamin B_1_, μg, *n* (%)				0.005
<1884.00	5,343 (49.99)	3,366 (48.89)	1977 (52.70)	
≥1884.00	4,477 (50.01)	2,922 (51.11)	1,555 (47.30)	
Vitamin B_2_, μg, *n* (%)				<0.001
<2464.00	5,582 (49.97)	3,446 (48.19)	2,136 (54.33)	
≥2464.00	4,238 (50.03)	2,842 (51.81)	1,396 (45.67)	
Vitamin B_6_, μg, *n* (%)				<0.001
<2455.00	5,340 (49.99)	3,293 (47.90)	2047 (55.09)	
≥2455.00	4,480 (50.01)	2,995 (52.10)	1,485 (44.91)	
Vitamin B_12_, μg, *n* (%)				<0.001
<6.99	5,415 (49.98)	3,344 (48.20)	2071 (54.34)	
≥6.99	4,405 (50.02)	2,944 (51.80)	1,461 (45.66)	
Vitamin C, mg, *n* (%)				<0.001
<89.40	5,132 (49.97)	3,137 (47.77)	1995 (55.37)	
≥89.40	4,688 (50.03)	3,151 (52.23)	1,537 (44.63)	
Vitamin D, μg, *n* (%)				<0.001
<6.60	5,256 (49.66)	3,207 (46.97)	2049 (56.26)	
≥6.60	4,564 (50.34)	3,081 (53.03)	1,483 (43.74)	
Vitamin E, mg, *n* (%)				<0.001
<7.48	5,404 (49.94)	3,314 (47.63)	2090 (55.59)	
≥7.48	4,416 (50.06)	2,974 (52.37)	1,442 (44.41)	
Vitamin K, μg, *n* (%)				<0.001
<82.70	5,345 (49.98)	3,261 (47.61)	2084 (55.80)	
≥82.70	4,475 (50.02)	3,027 (52.39)	1,448 (44.20)	
Alpha-carotene, μg, Mean ± S. E	467.94 ± 24.48	501.15 ± 27.76	386.57 ± 24.84	<0.001
Beta-carotene, μg, Mean ± S. E	2574.78 ± 92.75	2763.62 ± 116.01	2112.12 ± 102.61	<0.001
Beta-cryptoxanthin, μg, Mean ± S. E	89.33 ± 2.28	88.53 ± 2.86	91.30 ± 4.93	0.652
Lycopene, μg, Mean ± S. E	5445.93 ± 159.91	5443.55 ± 188.35	5451.76 ± 215.51	0.974
Lutein+ zeaxanthin, μg, Mean ± S. E	1956.90 ± 78.89	2083.75 ± 96.07	1646.11 ± 113.30	0.003

PIR, poverty-income ratio; BMI, body mass index; MET, metabolic equivalent; CVD, cardiovascular disease; WBC, white blood cell.

### Single vitamin exposure and periodontitis risk

The relationship between single dietary vitamin intake and periodontitis is presented in Table 2. In the weighted univariate logistic regression model (Model 1), higher level of nine dietary vitamins were all inversely associated with the odds of periodontitis risk. After adjusting age, gender, race/ethnicity, educational level, marital status, PIR, smoking status, anti-infectives, serum vitamin D, WBC, decayed teeth, dental floss, we found that higher intake levels of vitamin B_6_ (OR = 0.82, 95%CI: 0.72–0.94, *p* = 0.009) and vitamin E (OR = 0.79, 95%CI: 0.69–0.92, *p* = 0.004) were related to a decreased likelihood of developing periodontitis compared to lower intake levels, respectively.

**Table 2:** Single dietary vitamin intake and periodontitis risk.

Variables	Model 1		Model 2	
	OR (95%*CI*)	*p*	OR (95%CI)	*p*
Vitamin A, μg				
<528.00	Ref		Ref	
≥528.00	0.74 (0.65–0.83)	<0.001	0.88 (0.76–1.02)	0.094
Vitamin B_1_, μg				
<1884.00	Ref		Ref	
≥1884.00	0.86 (0.78–0.95)	0.005	0.94 (0.83–1.06)	0.323
Vitamin B_2_, μg				
<2464.00	Ref		Ref	
≥2464.00	0.78 (0.70–0.88)	<0.001	0.89 (0.77–1.03)	0.132
Vitamin B_6_, μg				
<2455.00	Ref		Ref	
≥2455.00	0.75 (0.67–0.84)	<0.001	0.82 (0.72–0.94)	0.009
Vitamin B_12_, μg				
<6.99	Ref		Ref	
≥6.99	0.78 (0.71–0.86)	<0.001	0.93 (0.82–1.04)	0.209
Vitamin C, mg				
<89.40	Ref		Ref	
≥89.40	0.74 (0.66–0.82)	<0.001	0.93 (0.82–1.04)	0.218
Vitamin D, μg				
<6.60	Ref		Ref	
≥6.60	0.69 (0.62–0.77)	<0.001	0.87 (0.76–1.00)	0.054
Vitamin E, mg				
<7.48	Ref		Ref	
≥7.48	0.73 (0.63–0.84)	<0.001	0.79 (0.69–0.92)	0.004
Vitamin K, μg				
<82.70	Ref		Ref	
≥82.70	0.72 (0.62–0.84)	<0.001	0.87 (0.73–1.04)	0.128

OR, odds ratio; CI, confidence interval. Model 1 was crude model; Model 2 was adjusted for age, gender, race/ethnicity, educational level, marital status, poverty-income ratio, smoking status, anti-infectives, serum vitamin D, white blood cell, decayed teeth, dental floss.

### Multi-vitamin exposures and periodontitis risk

**Figure 2:**
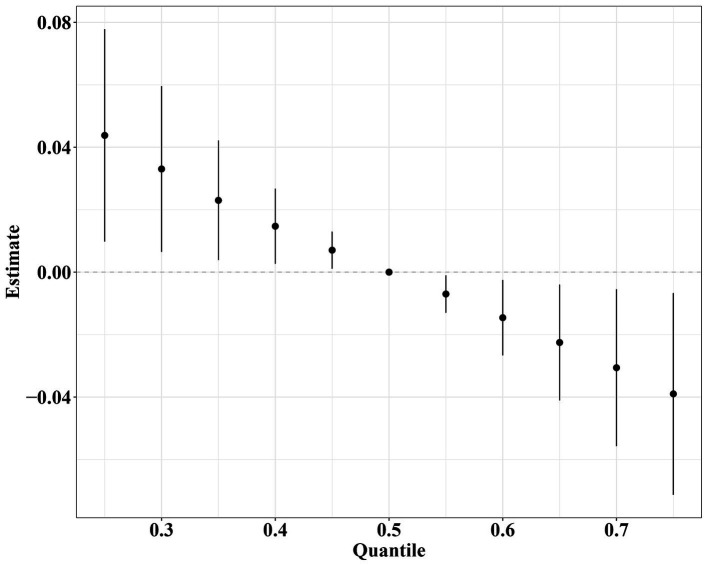
Combined effects of nine dietary vitamins mixtures and periodontitis by BKMR analysis. Model was adjusted for age, gender, race/ethnicity, educational level, marital status, poverty-income ratio, smoking status, anti-infectives, serum vitamin D, white blood cell, decayed teeth, and dental floss.

The BKMR model was employed to estimate the combined effect of nine dietary vitamins on periodontitis. In the fully adjusted BKMR model, the overall impact of nine dietary vitamins on periodontitis showed a downward trend, and an increase in the total level of vitamins mixture was associated with a decreased risk of periodontitis (Figure 2). Supplementary Figure S1 reveals the correlation of nine dietary vitamins. Vitamin B_1_ exhibited correlations with vitamin B_2_ (*r* = 0.87), vitamin B_6_ (*r* = 0.75), and vitamin B_12_ (*r* = 0.56). Additionally, notable correlations were observed between vitamin D and vitamin B_12_ (*r* = 0.55) as well as between vitamin D and vitamin B_2_ (*r* = 0.53), a substantial association was found between vitamin B_6_ and both vitamin B_2_ (*r* = 0.79) and vitamin B_12_ (*r* = 0.64). Lastly, vitamin E and vitamin K was correlated as well (*r* = 0.53). The nine dietary vitamins were grouped based on the correlations analysis. The GroupPIP and CondPIP derived from the BKMR model for nine dietary vitamins are summarized in Supplementary Table S3. The GroupPIP of group four (vitamin E and K: 1) was higher than other three groups (vitamin A: 0.302; vitamin B_1_, B_2_, B_6,_ B_12_ and D: 0.292; vitamin C: 0.226). Vitamin E (CondPIP = 0.998) contributed most to the BKMR model for the periodontitis risk. Additionally, when all remaining vitamins were at their median levels, the periodontitis risk decreased with increased concentration levels of vitamin E and vitamin B_2_ (Supplementary Figure S2).

As shown in Table 3, WQS regression analysis indicated a negative association between nine dietary vitamins co-exposure and periodontitis risk (OR = 0.88, 95%CI: 0.81–0.95, *p* = 0.001). The estimated weights of nine dietary vitamins for the WQS model was calculated (Supplementary Figure S3). The highest weighted chemical in the WQS model was vitamin E, followed by vitamin B_12_ and vitamin D.

**Table 3:** The combined effect of nine dietary vitamins on periodontitis by WQS model.

Model	OR (95% CI)	*p*
WQS model	0.88 (0.81–0.95)	0.001

WQS, weighted quantile sum; OR, odds ratio, CI, confidence interval; Model was adjusted for age, gender, race/ethnicity, educational level, marital status, poverty-income ratio, smoking status, anti-infectives, serum vitamin D, white blood cell, decayed teeth, dental floss.

Similar to the WQS model, an increase in the qgcomp index was related to decreased risk of periodontitis (Table 4, OR = 0.90, 95%CI: 0.84–0.96, *p* = 0.001). In the qgcomp model, vitamin E received the highest negative weights for the periodontitis risk, followed by vitamin B_2_ and vitamin D, respectively (Supplementary Figure S4).

**Table 4:** The combined effect of nine dietary vitamins on periodontitis by qgcomp model.

Model	OR (95% CI)	*p*
Qgcomp model	0.90 (0.84–0.96)	0.001

Qgcomp, Quantile g-computation; OR, odds ratio, CI, confidence interval; Model was adjusted for age, gender, race/ethnicity, educational level, marital status, poverty-income ratio, smoking status, anti-infectives, serum vitamin D, white blood cell, decayed teeth, dental floss.

## Discussion

In the present study, we observed that dietary vitamin B_6_ and vitamin E were associated with decreased odds of periodontitis, respectively. Furthermore, we used three statistical approaches, namely BKMR, WQS regression, and qgcomp model, to evaluate the associations of nine dietary vitamins mixtures with periodontitis risk. Three models suggested that the mixtures of nine dietary vitamins showed inverse overall associations with periodontitis risk, and vitamin E made the most contribution in the association between nine dietary vitamins mixtures and periodontitis risk.

Consistent with previous research findings (8, 17), this study indicated a correlation between higher intakes of vitamins B_6_ and E and a reduced risk of periodontal, respectively. Some studies have indicated that an excessive formation of reactive oxygen species (ROS) occurs in the periodontal tissues, leading to a reduction in antioxidant capacity (18). Oxidative stress is believed to play a role in the pathogenesis of periodontitis (19). Vitamin E, as an antioxidant, can effectively eliminate ROS and contribute significantly to the repair of damaged cells (20). Furthermore, vitamin E has been reported to exert antioxidant effects by reducing lipid peroxidation and increasing the level of superoxide dismutase (21, 22). Given that chronic inflammation, mediated by immune cells, is accountable for the degradation of periodontal tissue, the anti-inflammatory properties of vitamin E could significantly inhibit the expression of inducible nitric oxide synthase (iNOS) in periodontal tissues, reduce the levels of gingival fibroblast production of [interleukin (IL)-1β and IL-6], and increase the levels of human β-defensins, which may help improve host anti-inflammatory environment and maintain oral health (8, 23–25).

Nevertheless, after controlling for all potential confounding factors, no evidence of significant associations between vitamin A, vitamin B_1_, vitamin B_2_, vitamin B_12_, vitamin C, vitamin D, and vitamin K and periodontitis risk was found in our current investigation, which is inconsistent with previous studies that reported these dietary vitamins and periodontitis (6, 10, 26). We speculated that the observed association may be to differences in sample size and adjusted confounding variables. Prospective studies with large sample size are warranted to further verify the relationship of single dietary vitamin and periodontitis.

Due to the diverse nutritional composition of food, individuals are simultaneously exposed to multiple vitamins in their daily life, an increasing number of researchers are advocating to explore the relationship of mixed vitamin exposures and human health (27). In general, BKMR, WQS regression, and qgcomp models are commonly employed to estimate the overall impact of mixed chemical exposure (28, 29). For instance, a cross-sectional study conducted by Tang et al., they found that multivitamins mixed intake was negatively associated with the obesity risk among children and adolescents via using the BKMR analysis (12). In the study of Peng et al., WQS regression model indicated an association of serum multivitamin levels and reduced risk of non-alcoholic fatty liver disease (30). To our knowledge, there is little evidence to present the association between mixed dietary vitamins and periodontitis risk so far. Therefore, in this study we used three methods to capture the association between mixed dietary vitamins and periodontitis risk the general population of the U.S. Remarkably, the findings of BKMR, WQS regression, and qgcomp models consistently revealed that mixture of vitamin A, vitamin B_1_, vitamin B_2_, vitamin B_6_, vitamin B_12_, vitamin C, vitamin D, vitamin E, and vitamin K had a significant negative combined effect on the risk of periodontitis. In addition, we also found that vitamin E contributes greatly and negatively to the association of mixed dietary vitamins and periodontitis. These results also indicated that adequate intake of vegetables, lean meat, milk, eggs, etc. into our daily diet can effectively mitigate the development of periodontitis. However, the possible mechanism related to the overall effect between mixed dietary vitamins and periodontitis remains unclear. Further exploration is needed regarding the potential mechanisms in the association.

The present study possesses several advantages. Firstly, this is the first study to explore the joint impact of dietary vitamins mixtures on periodontitis among adults in the United States, which might provide significant evidence for exploring the effects of dietary vitamins on periodontitis. Secondly, three different statistical methods were adopted to assess the relationship association between nine dietary vitamins mixtures and periodontitis risk. The study, however, does possess certain limitations that must be acknowledged. Firstly, the cross-sectional study design precludes us from establishing a definitive causal relationship between dietary vitamins and the risk of periodontitis. Secondly, dietary vitamins intake in this study was based on 24 h dietary recall interviews, and thereby there may be an inherent bias. Lastly, NHANES database lacked the information on the specific forms of vitamins B1, B2, B6, B12, C, D, E and K. Further prospective studies are needed to verify our findings and clarify the underlying mechanisms.

## Conclusion

In summary, dietary vitamin B_6_ and vitamin E were found to be associated with decreased odds of periodontitis, respectively. Additionally, the mixture-exposed analyses consistently showed the negative correlations between nine dietary vitamins mixtures and periodontitis, with a highest contribution vitamin E towards the combined effect. Our findings provide evidence for exploring the effects of multiple dietary vitamins exposures on periodontitis.

## Data availability statement

Publicly available datasets were analyzed in this study. This data can be found here: NHANES database, https://wwwn.cdc.gov/nchs/nhanes/.

## Ethics statement

The requirement of ethical approval was waived by Department of Stomatology, Liuzhou People’s Hospital for the studies involving humans because the data was accessed from a publicly available database. The studies were conducted in accordance with the local legislation and institutional requirements. The ethics committee/institutional review board also waived the requirement of written informed consent for participation from the participants or the participants’ legal guardians/next of kin because retrospective nature of the study.

## Author contributions

FL: Conceptualization, Formal analysis, Project administration, Supervision, Writing – original draft, Writing – review & editing. ML: Data curation, Formal analysis, Investigation, Methodology, Writing – review & editing. YZ: Data curation, Formal analysis, Investigation, Methodology, Writing – review & editing.
