# Supplementary material for: Associations between single and multiple dietary vitamins and the risk of periodontitis: results from NHANES 2009–2014
Source: Front Nutr. 2024 Apr 8;11:1347712. doi: 10.3389/fnut.2024.1347712 (PMC11033469; doi:10.3389/fnut.2024.1347712)
Supplement: Supplementary file 1 [file Table_1.DOCX]

**Supplemental Table 1 Sensitivity analysis of data before and after interpolation**

| **Variables** | **After** | **Before** | **Statistics** | ***P*** |
| --- | --- | --- | --- | --- |
| BMI, kg/m^2^, Mean ± S. E | 29.17 ± 0.13 | 29.16 ± 0.12 | t = -0.829 | 0.411 |
| WBC, 1000 cells/uL, Mean ± S. E | 7.13 ± 0.04 | 7.13 ± 0.04 | t = -0.232 | 0.818 |
| Serum vitamin D, nmol/L, Mean ± S. E | 71.47 ± 0.91 | 71.53 ± 0.92 | t = 1.017 | 0.314 |

BMI, body mass index; WBC, white blood cell.

**Supplemental Table 2 Selection of covariates**

| **Variables** | **OR (95% CI)** | ***P*** |
| --- | --- | --- |
| Age | 1.01 (1.00-1.01) | 0.010 |
| Gender |  |  |
| Female | Ref |  |
| Male | 1.99 (1.83-2.16) | <0.001 |
| Race/ethnicity |  |  |
| White | Ref |  |
| Black | 2.81 (2.31-3.41) | <0.001 |
| Other | 2.13 (1.74-2.60) | <0.001 |
| Education level |  |  |
| Less than high school | Ref |  |
| High school or above | 0.37 (0.32-0.44) | <0.001 |
| Marital status |  |  |
| Married & Living with partner | Ref |  |
| Never married &Divorced &Separated &Widowed | 1.44 (1.26-1.63) | <0.001 |
| PIR |  |  |
| <1 | Ref |  |
| ≥1 | 0.42 (0.35-0.49) | <0.001 |
| Unknown | 0.68 (0.54-0.86) | 0.002 |
| Smoking status |  |  |
| Never smoked | Ref |  |
| Former smoker | 1.30 (1.09-1.55) | 0.005 |
| Current smoker | 3.04 (2.61-3.54) | <0.001 |
| Drinking status |  |  |
| Never drinker | Ref |  |
| Moderate drinker | 0.93 (0.70-1.24) | 0.628 |
| Heavy drinker | 1.04 (0.85-1.28) | 0.689 |
| Unknown | 1.43 (1.16-1.77) | 0.002 |
| Physical activity |  |  |
| <450 | Ref |  |
| ≥450 | 0.85 (0.76-0.94) | 0.003 |
| Hypertension |  |  |
| No | Ref |  |
| Yes | 1.35 (1.19-1.54) | <0.001 |
| Diabetes |  |  |
| No | Ref |  |
| Yes | 1.63 (1.38-1.93) | <0.001 |
| Dyslipidemia |  |  |
| No | Ref |  |
| Yes | 1.03 (0.89-1.19) | 0.710 |
| CVD |  |  |
| No | Ref |  |
| Yes | 1.11 (0.96-1.29) | 0.156 |
| Nonsteroidal anti-inflammatory agents |  |  |
| No | Ref |  |
| Yes | 1.09 (0.93-1.29) | 0.296 |
| Anti-infectives |  |  |
| No | Ref |  |
| Yes | 0.74 (0.57-0.97) | 0.033 |
| BMI |  |  |
| <25 | Ref |  |
| 25-30 | 1.16 (1.02-1.31) | 0.030 |
| ≥30 | 1.39 (1.21-1.60) | <0.001 |
| Serum vitamin D |  |  |
| <50 | Ref |  |
| ≥50 | 0.50 (0.43-0.57) | <0.001 |
| WBC | 1.12 (1.09-1.16) | <0.001 |
| Decayed teeth |  |  |
| No | Ref |  |
| Yes | 3.09 (2.68-3.56) | <0.001 |
| Dental floss |  |  |
| No | Ref |  |
| Yes | 0.50 (0.45-0.56) | <0.001 |
| Total energy | 1.00 (1.00-1.00) | <0.001 |
| Total sugars | 1.00 (1.00-1.00) | 0.003 |

OR, odds ratio, CI, confidence interval; PIR, poverty-income ratio; BMI, body mass index; CVD, cardiovascular disease; WBC, white blood cell.

**Supplemental Table 3 GroupPIP and CondPIP of nine dietary vitamins**

| **Variables** | **Group** | **GroupPIP** | **CondPIP** |
| --- | --- | --- | --- |
| Vitamin A | 1 | 0.302 | 1 |
| Vitamin B_1_ | 2 | 0.292 | 0.075 |
| Vitamin B_2_ | 2 | 0.292 | 0.610 |
| Vitamin B_6_ | 2 | 0.292 | 0.096 |
| Vitamin B_12_ | 2 | 0.292 | 0.075 |
| Vitamin D | 2 | 0.292 | 0.144 |
| Vitamin C | 3 | 0.226 | 1 |
| Vitamin E | 4 | 1 | 0.998 |
| Vitamin K | 4 | 1 | 0.002 |

GroupPIP =Group posterior inclusion probability; CondPIP =Conditional posterior inclusion probability.
