# Supplementary figures and images for: Associations between single and multiple dietary vitamins and the risk of periodontitis: results from NHANES 2009–2014
Source: Front Nutr. 2024 Apr 8;11:1347712. doi: 10.3389/fnut.2024.1347712 (PMC11033469; doi:10.3389/fnut.2024.1347712)

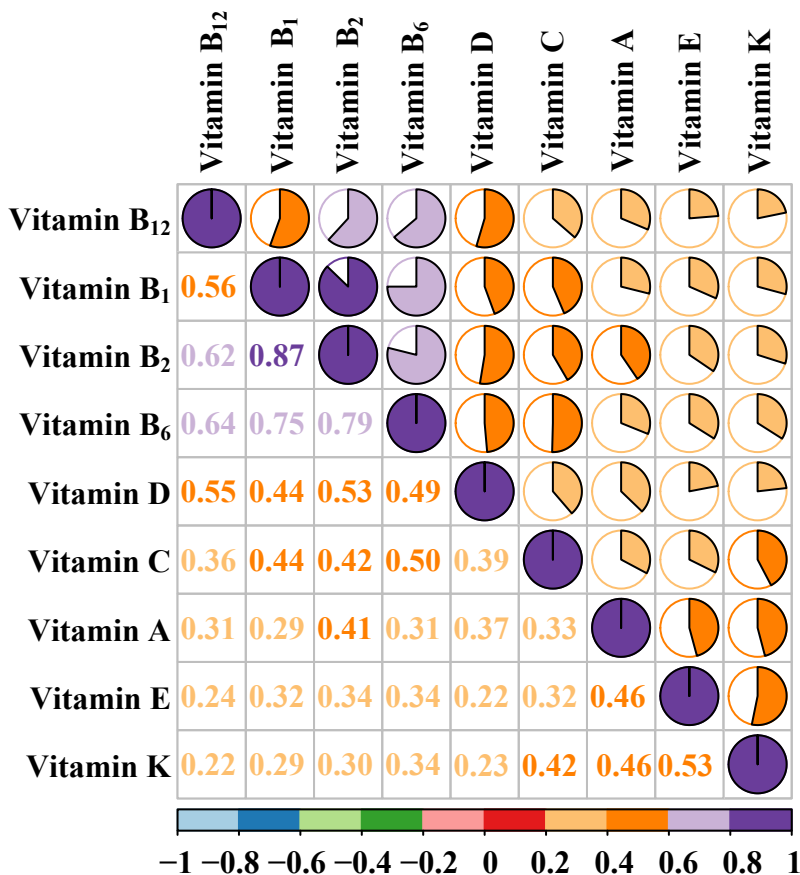

Supplement: Supplementary file 2 [file Image_1.pdf]

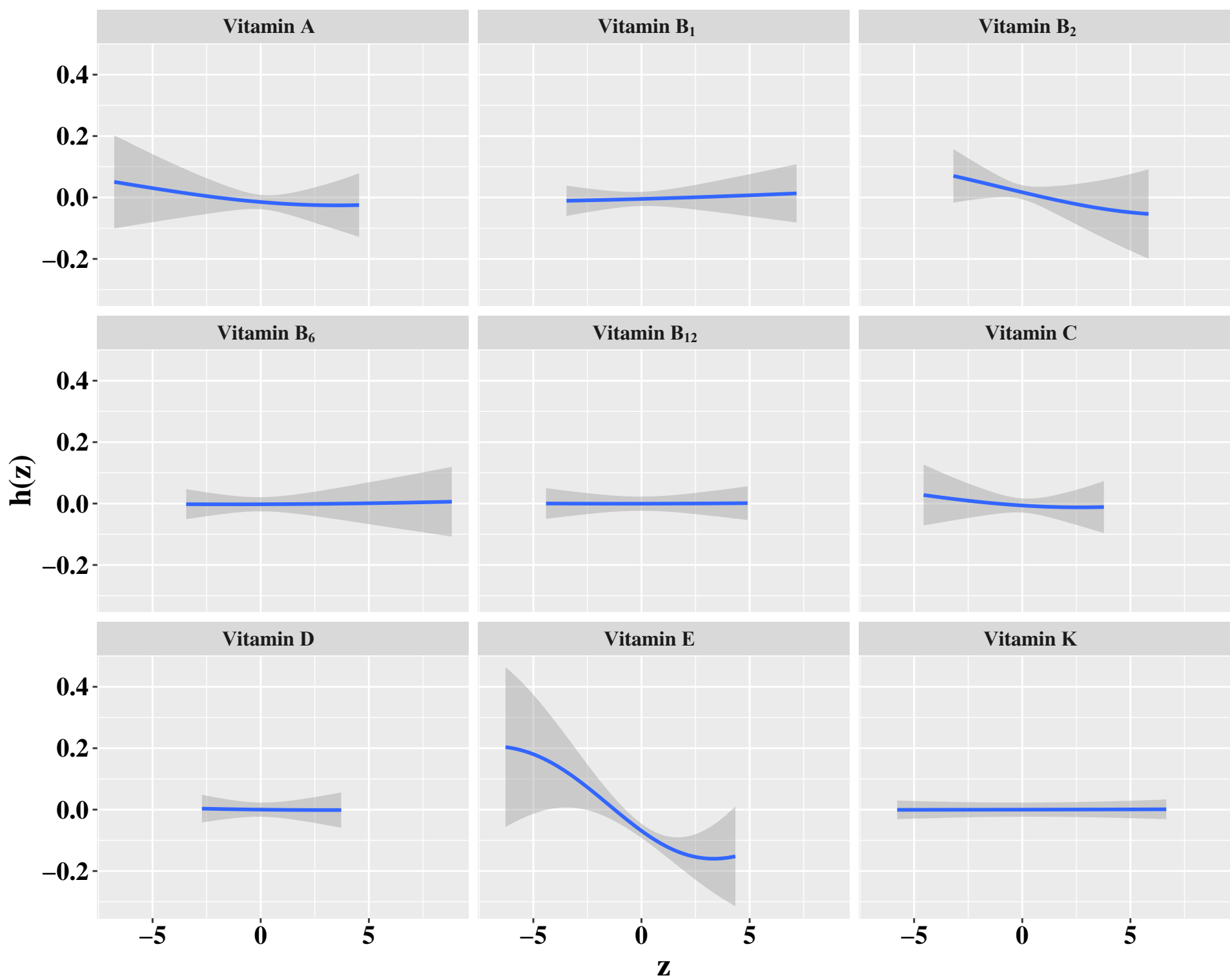

Supplement: Supplementary file 3 [file Image_2.pdf]

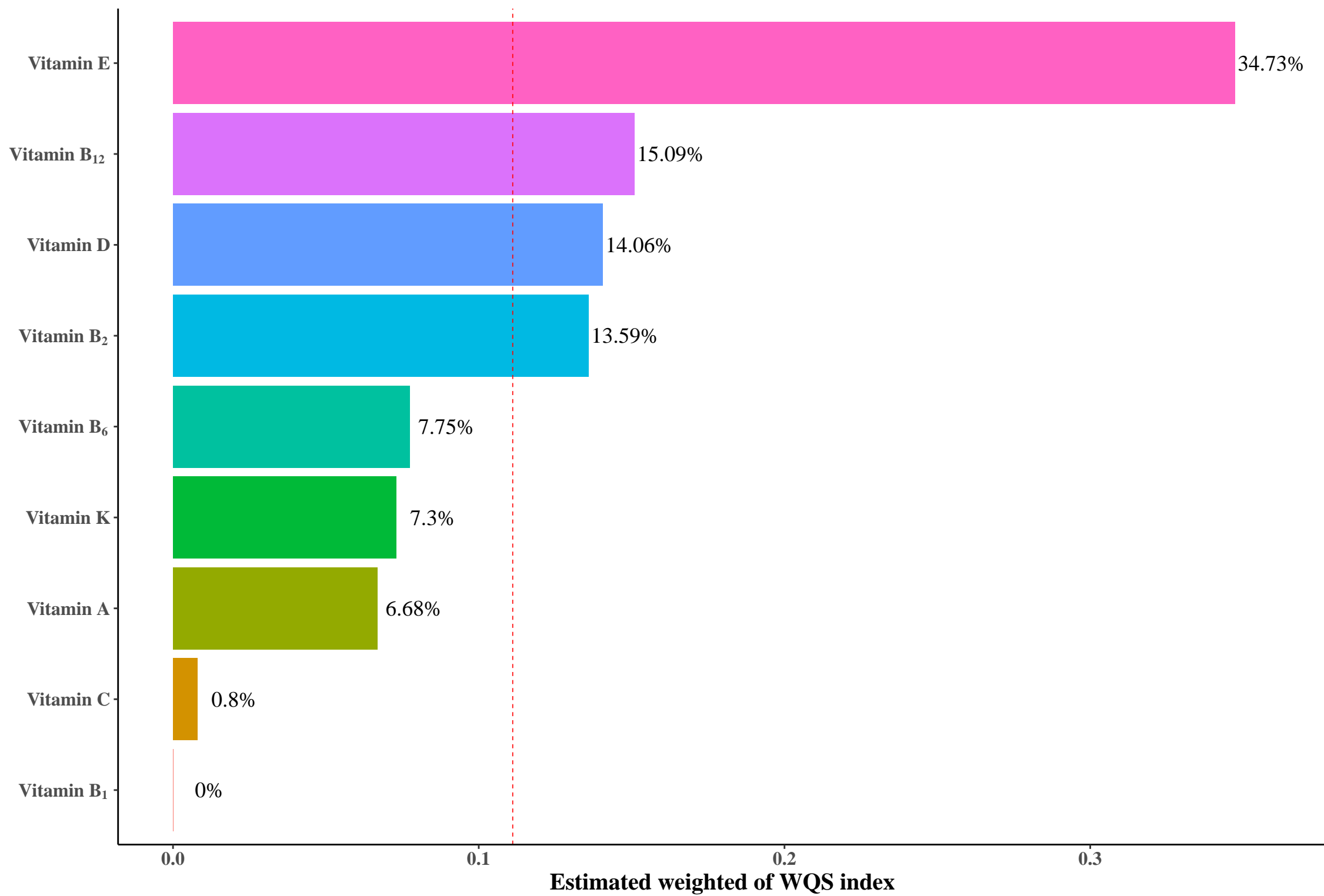

Supplement: Supplementary file 4 [file Image_3.pdf]

Variable

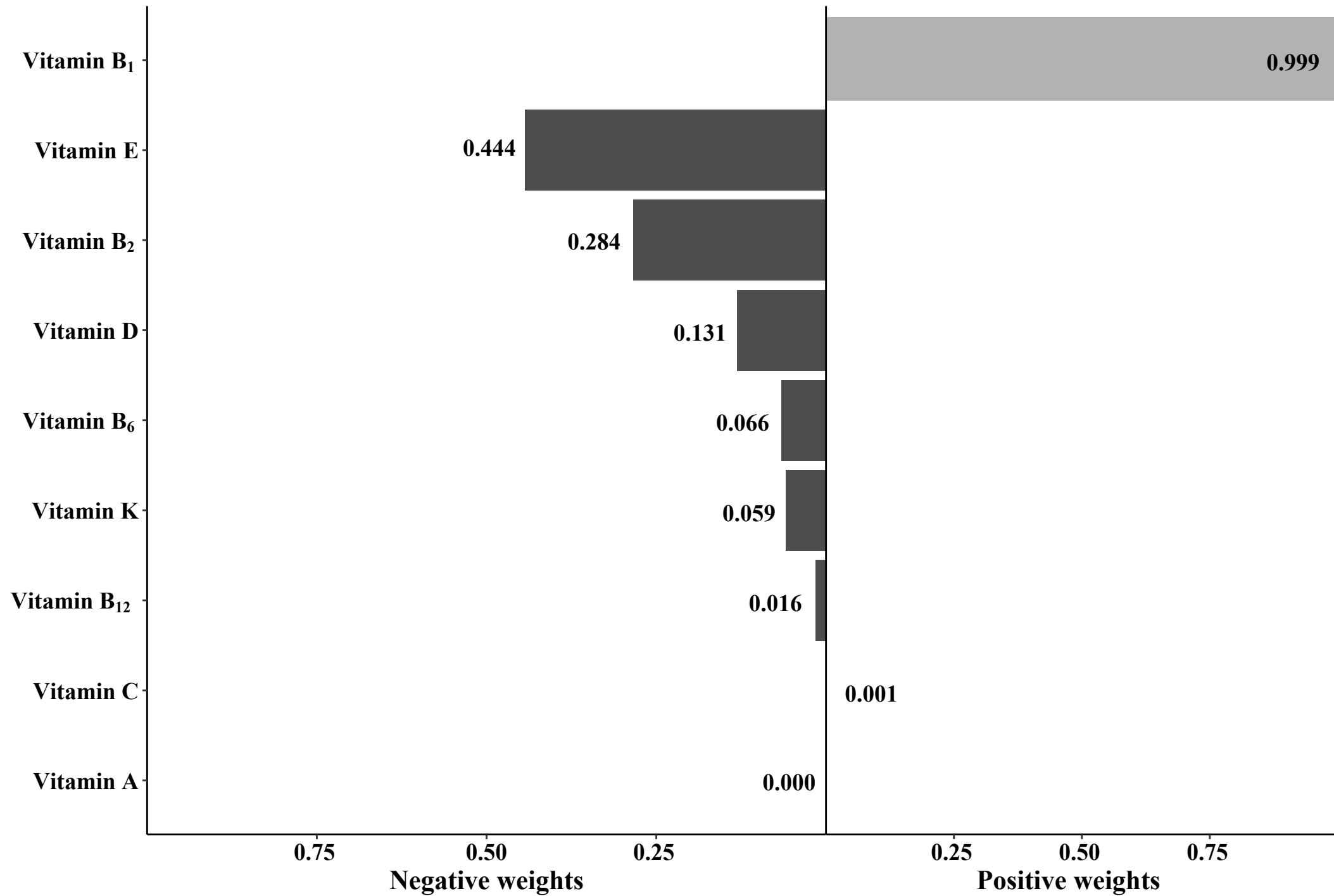

Supplement: Supplementary file 5 [file Image_4.pdf]
